# Outcomes of the Deloyers procedure: A systematic review and meta‐analysis of proportions

**DOI:** 10.1111/codi.70346

**Published:** 2025-12-22

**Authors:** Lucas Monteiro Delgado, Gabriel Leal Barone, Giulia Luiza Garcia, Henrique Vaz da Mota, Giovanna Barbaroto Pilon, Lucas Soares de Souza Pinto Guedes, Sérgio Mazzola Poli de Figueiredo, Bernardo Fontel Pompeu, Fernanda Bellotti Formiga

**Affiliations:** ^1^ Universidade Federal de Minas Gerais (UFMG) Belo Horizonte MG Brazil; ^2^ University of São Caetano Do Sul São Caetano do Sul Brazil; ^3^ University of North Carolina at Chapel Hill Chapel Hill North Carolina USA; ^4^ Surgery Department Hospital Heliópolis São Paulo SP Brazil

**Keywords:** colorectal anastomosis, Deloyers procedure, functional outcomes, left hemicolectomy, systematic review

## Abstract

**Background:**

Extended left hemicolectomies may result in a short transverse colon and excessive mesenteric tension, precluding a tension‐free anastomosis. The Deloyers procedure is a colonic rotation technique that facilitates anastomosis without total colectomy. This study aimed to evaluate short‐term outcomes following the Deloyers procedure in patients undergoing left‐sided colorectal resections.

**Methods:**

A systematic review and meta‐analysis were conducted using PubMed, Scopus and the Cochrane Central Register to identify observational studies published up to March 2025. Continuous outcomes were pooled as means with 95% confidence intervals (CIs) and binary outcomes as proportions. Heterogeneity was assessed using the *I* ^2^ statistic and Cochrane *Q*‐test. All analyses were performed using R (v4.4.1).

**Results:**

Thirteen studies including 287 patients undergoing the Deloyers procedure were analysed. The pooled mean operating time was 256.7 min (95% CI, 188.5–349.6), intraoperative blood loss was 327.5 mL (95% CI, 227.9–470.6) and the perioperative transfusion rate was 25% (95% CI, 16%–35%). Postoperative recovery outcomes included a mean hospital stay of 9.9 days (95% CI, 7.6–12.9), an ileus rate of 15% (95% CI, 7%–30%) and an average of 2.9 bowel movements per day (95% CI, 2.4–3.5). Reported complications included Clavien–Dindo grade I–II events in 36% of patients (95% CI, 14%–66%) and grade III–IV events in 12% (95% CI, 5%–24%). The pooled rate of surgical site infection was 13% (95% CI, 4%–34%), small bowel obstruction 6% (95% CI, 0%–35%) and anastomotic leakage 1% (95% CI, 0%–7%). The overall mortality rate was 0% (95% CI, 0%–1%), with two deaths reported in patients with significant comorbidities and advanced oncological disease.

**Conclusion:**

The Deloyers procedure is a feasible surgical alternative in patients requiring extended left‐sided colectomy, providing acceptable operative times, functional recovery and complication rates. It may help avoid total colectomy in anatomically complex cases.

**What does this paper add to the literature?**

This study provides a quantitative synthesis of Deloyers procedure outcomes by pooling 287 patients across 13 series. It defines benchmark perioperative and functional results, shows low leak and mortality with acceptable morbidity and refines surgical decision‐making by supporting colon‐preserving reconstruction in anatomically challenging left‐sided colorectal resections.

## INTRODUCTION

**FIGURE 1:**
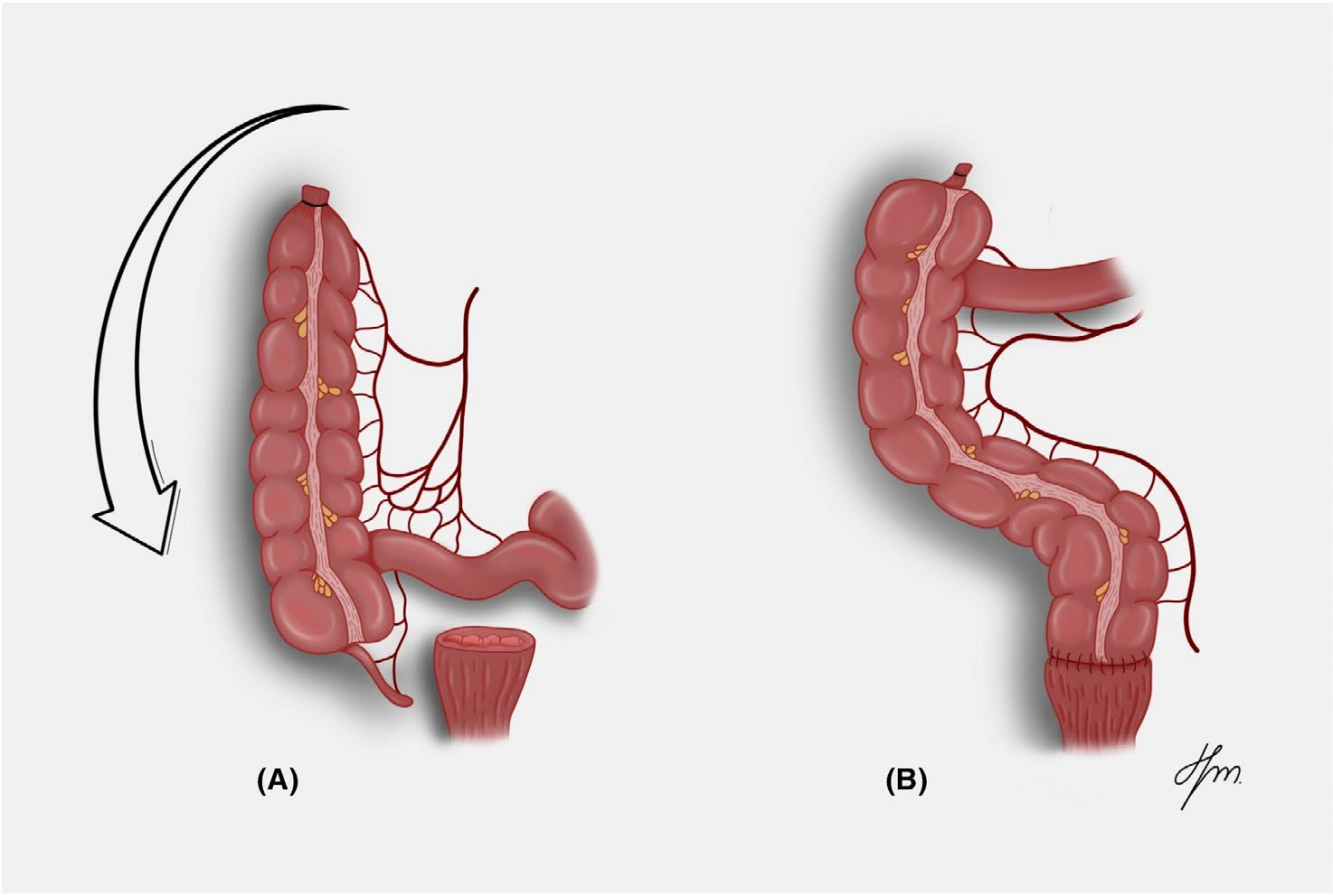
Deloyers procedure. (A) The right colon is fully mobilized with preservation of the ileocolic pedicle and release of the hepatic flexure, prepared for rotation around the ileocolic pedicle. (B) Tension‐free colorectal anastomosis with a straight, untwisted mesentery after transposition of the right colon.

The Deloyers reconstruction technique is a surgical procedure used to restore bowel continuity after extended left hemicolectomy or subtotal colectomy, particularly when the remaining right colon cannot reach the rectal stump without tension. The procedure involves mobilization of the right colon with preservation of the ileocolic artery, followed by rotation around the ileocolic pedicle to achieve a tension‐free colorectal or coloanal anastomosis (Figure 1) [1]. This approach preserves the ileocaecal valve and a segment of the right colon, which can improve stool consistency and reduce bowel frequency compared with ileorectal anastomosis, where the ileum is directly anastomosed to the rectum [1, 2, 3].

The Deloyers procedure is indicated when standard colorectal anastomosis is not feasible due to insufficient colonic length or vascular supply after extensive left‐sided colonic resection. Clinical series have reported low morbidity, acceptable anastomotic complication rates and improved functional outcomes [4], including better stool consistency, fewer bowel movements and overall quality of life compared with ileorectal anastomosis [3]. Despite these encouraging results, most available evidence comes from small retrospective cohorts or single‐institution experiences, often involving heterogeneous patient populations and variable follow‐up durations. Given the increasing use of this technique in complex colorectal surgery, a more robust appraisal of outcomes is warranted.

Therefore, we conducted a systematic review and single‐arm meta‐analysis to synthesize the existing literature on the Deloyers procedure. Our objectives were to estimate perioperative morbidity, anastomotic complication rates, long‐term functional outcomes and overall safety, thereby providing a more comprehensive evidence base to guide clinical decision‐making in patients requiring this complex reconstruction.

## METHODS

### Protocol and registration

We performed the systematic review and meta‐analysis according to the Cochrane Handbook for Systematic Reviews of Interventions and structured it according to the Preferred Reporting Items for Systematic Reviews and Meta‐Analysis (PRISMA) guidelines, presented in Table S1 [5, 6]. The study protocol was registered in the International Prospective Register of Systematic Reviews (PROSPERO) under registration number CRD420251135524 [7].

### Outcomes

The outcomes of interest were: (1) intraoperative blood loss; (2) blood transfusion; (3) operating time; (4) hospital stay; (5) ileus; (6) bowel movements; (7) Clavien–Dindo (CD) grade I–II complications; (8) CD grade III–IV complications; (9) small bowel obstruction; (10) surgical site infection (SSI); (11) mortality.

### Eligibility criteria

Inclusion in this meta‐analysis was limited to studies that met all the following eligibility criteria: (1) enrolled adults (≥18 years old) undergoing extended left colectomy with Deloyers reconstruction and (2) reported at least one of the outcomes of interest. Studies were excluded if they met any of the following criteria: (1) included other reconstruction techniques without stratified outcome reporting; (2) were purely technical descriptions; (3) lacked full‐text availability; or (4) were case reports, trial registrations without available results, meta‐analyses, reviews or animal studies.

### Search strategy and study selection

We systematically searched PubMed, Embase and Cochrane Library databases from inception to 23 November 2025. The detailed search strategy for each database is provided in Table S2. We also searched the references of the included studies and previous systematic reviews and meta‐analyses aiming for the inclusion of additional studies [8].

Two authors (G.L.B. and G.L.G.) independently conducted the search, imported results into Rayyan, a web‐based systematic review tool and triaged the studies. After the exclusion of duplicates and titles/abstracts unrelated to the clinical question, the eligibility of each remaining study was assessed based on the review of the full‐text articles. Disagreements were resolved by consensus.

### Data extraction

Two authors (G.L.B. and G.L.G.) independently extracted data from the included studies using a standardized form. Extracted information included: (1) general study data (first author, year of publication, country and study design); (2) sample characteristics (number of patients, number and percentage of males, mean age, body mass index (BMI), American Society of Anesthesiologists (ASA) physical status score, and follow‐up time in months) and (3) surgical characteristics (surgical approach, type of anastomosis and clinical indication).

### Risk of bias and quality assessment

Two independent reviewers (G.L.B. and G.L.G.) assessed the risk of bias using the Joanna Briggs Institute (JBI) Critical Appraisal Checklist for Case Series [9]. Disagreements were resolved through consensus. Funnel plots were not performed because they are not recommended for single‐arm meta‐analyses, as the relationship between study size and effect is unclear and these methods are unreliable for detecting publication bias in meta‐analyses of proportions [10, 11].

### Statistical analysis

Pooled proportions of outcomes were calculated using the inverse variance method, applying logit or Freeman‐Tukey double arcsine transformations as appropriate. Between‐study heterogeneity was assessed using the Cochran *Q* test and the *I* ^2^ statistic, with heterogeneity considered significant when *p* < 0.10 and *I* ^2^ > 25%. In the presence of significant heterogeneity, Baujat plots were generated to evaluate the influence of each study on the overall effect size and variability [12]. In addition, a leave‐one‐out sensitivity analysis was performed by sequentially excluding each study from the meta‐analysis to assess the stability of the results and ensure that findings were not driven by any single study. All statistical analyses were performed using R statistical software (version 4.5; R Foundation for Statistical Computing, Vienna, Austria).

## RESULTS

### Study selection

**FIGURE 2:**
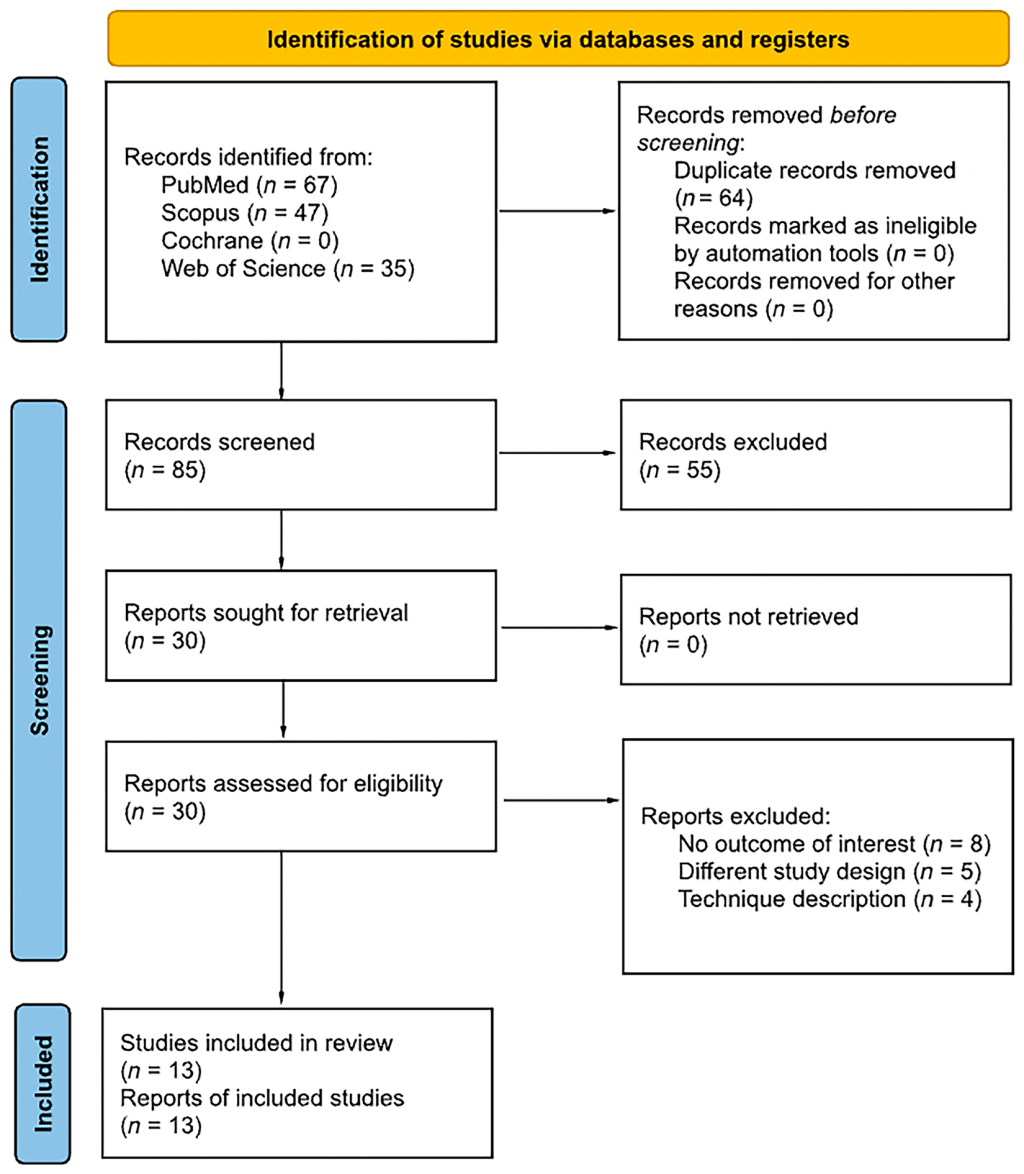
PRISMA flow diagram.

As detailed in Figure 2, the initial search identified 149 results. After removal of duplicate records and assessment of the studies based on title and abstract, 30 full‐text studies remained for full review according to prespecified criteria. Of these, 13 studies were included [1, 2, 3, 4, 13, 14, 15, 16, 17, 18, 19, 20, 21].

### Patient characteristics

The cohort comprised 287 patients who underwent the Deloyers procedure. Approximately 57% were male, and the mean age across studies ranged from 41.7 to 74.3 years. The distribution of perioperative physical status was comparable between ASA I–II (47.9%) and ASA III–IV (52.1%). Reported follow‐up ranged from 11 to 85 months, with most studies describing a mean or median duration longer than 12 months. General study characteristics and patient demographics are summarized in Table 1.

**TABLE 1:** General study characteristics and patient demographics.

Author	Country	No of patients	Design	Male sex, *n* (%)	Age (years), mean ± SD	BMI (kg/m^2^), mean ± SD	ASA I‐II, *n* (%)	ASA III‐IV, *n* (%)	Follow‐up, mean or median ± SD
Carpinteyro‐Espín, 2023	NA	16	Obs	9 (56.2)	51	24.3	10 (62.5)	6 (37.5)	55 months^a^
Chen, 2020	China	4	Obs	2 (50)	64.5 ± 16.6	NA	0 (0)	4 (100)	15 months^b^
Choi, 2020	Korea	6	Obs	3 (50)	74.3 ± 5.7	24.3 ± 2.9	4 (66.6)	2 (33.3)	39 (range 10–74) months^a^
Dalmau, 2024	NA	10	Obs	NA	NA	NA	NA	NA	22.2 ± 9.8 months^b^
Dumont, 2013	NA	29	Obs	10 (25.6)	55.1 ± 11.7	24.3 ± 3.9	NA	NA	20 ± 27 months^c^
Eldein, 2024	Egypt	20	Obs	10 (50)	53.5 ± 14.4	NA	NA	NA	Minimal 12 months after procedure^d^
Kontovounisios, 2014	London	14	Obs	9 (64.2)	58.7 ± 8.7	28 ± 3.5	9 (64.3)	5 (35.7)	11 months^a^
Manceau, 2012	France	48	Obs	38 (79)	67 ± 10.2	24 ± 5.4	19 (40)	29 (60)	26 months^a^
Salgado‐Nesme, 2017	Brazil	11	Obs	7 (63)	41.7 ± 16.6	25.9 ± 8.3	7 (63)	4 (36)	18 (4–38) months^c^
Schabl, 2025	Austria	19	Obs	11 (58)	58 ± 17	26.5 ± 5	10 (57.9)	8 (42.1)	85 months^a^
Sciuto, 2016	Italy	10	Obs	8 (80)	58.8 ± 10.3	23.6 ± 2.2	7 (70)	3 (30)	Minimal 12 months after procedure^d^
Shariff, 2011	NA	3	Obs	1 (33)	45 ± 14.3	NA	NA	NA	41 months^b^
Sobrado, 2025	USA	97	Obs	50 (51.5)	58.8 ± 14	27.9 ± 6.1	30 (30.9)	67 (69.1)	82.2 months^b^

Abbreviations: ASA I–II, American Society of Anesthesiologists physical status I or II (low surgical risk); ASA III–IV, American Society of Anesthesiologists physical status III or IV (moderate to high surgical risk); BMI, body mass index; NA, not available or not reported; Obs, observational study; SD, standard deviation.

^a^ Follow‐up reported as median only.

^b^ Follow‐up reported as mean or median.

^c^ Follow‐up reported as range or interquartile interval.

^d^ Minimal follow‐up period reported without mean or median.

Among the reported intervention characteristics and outcomes, the surgical approach was predominantly laparoscopic (59%), followed by open procedures (39%) and robotic surgery (1%). The type of anastomosis was most often colorectal or colonic stapled (80%–90%), while hand‐sewn or Turnbull‐Cutait techniques accounted for 10%–20% of cases. Clinical indications for the Deloyers procedure varied. Colorectal cancer (CCR) was the most frequent indication, present in about 70% of studies. Other reported indications included diverticular disease in around 30% of cases, ischaemic colitis in approximately 20% and local recurrence in 25%. Surgical features and clinical indications of included patients are summarized in Table 2.

**TABLE 2:** Surgical features and clinical indications.

Author	Surgical approach	Type of anastomosis	Clinical indications for Deloyers
Carpinteyro‐Espín, 2023	NA	NA	BEN (75%)
Chen, 2020	Laparoscopic (100%)	Colorectal (50%), Coloanal (25%)	CCR (50%), RECUR, CPER
Choi, 2020	Laparoscopic (100%)	Colorectal (100%)	CCR (50%), Hartmann (50%)
Dalmau, 2024	Laparoscopic (majority)	Stapled Colorectal	Not specified
Dumont, 2013	NA	NA	FAA (45%), ISC (21%), RECUR (10%), CCR (7%), DIV (6%), SINC (7%)
Eldein, 2024	NA	NA	CCR (74.1%), CPER (34.8%), RECUR (43.8%), OUT (9.1%)
Kontovounisios, 2014	Open (100%)	Colorectal/Coloanal	CCR, DIV, ISC, FAA, SINC
Manceau, 2012	NA	Colorectal/Coloanal	Hartmann (35%)
Salgado‐Nesme, 2017	NA	Colorectal (90%), Coloanal (10%)	DIV (36%), APEND (9%), GSW (9%)
Schabl, 2025	Open (89.5%)	NA	CCR (15.8%), CSD (10.5%), DIV (42.1%), RET (10.5%), MES (5.3%), PERF (5.3%), GIST (5.3%)
Sciuto, 2016	Lap (90%), Open (10%)	Colorectal (100%)	CCR (40%), POL (10%), DIV (10%), ISC (10%), RET (20%), STR (20%)
Shariff, 2011	NA	Double‐stapled (100%)	CCR, Crohn
Sobrado, 2025	Open (82.5%), Laparoscopic (16.5%), Robotic (1%)	Stapled (85.6%), Handsewn (10.3%), Turnbull (3.1%)	CCR (50.5%), DIV (21.6%), Crohn (8.2%), ISC (9.3%), CONST (4.1%), Non‐CCR (3.1%), VOLV (1%)

Abbreviations: APEND, incidental appendicitis; BEN, benign disease (unspecified); CCR, colorectal cancer; CONST, chronic constipation; CPER, peritoneal carcinomatosis; Crohn, Crohn's disease; CSD, descending or sigmoid colon cancer; DIV, diverticular disease; FAA, failed anterior anastomosis; GIST, gastrointestinal stromal tumour; GSW, gunshot wound; Hartmann, Hartmann's procedure reversal; ISC, colonic ischaemia; MES, mesenteric infiltration; NA, not available; Non‐CCR, non‐colorectal cancer; OUT, other causes; PERF, colonic perforation; POL, colorectal polyps; RECUR, local cancer recurrence; RET, rectal cancer; SINC, synchronous colorectal cancer; STR, anastomotic stricture; VOLV, sigmoid volvulus.

### Pooled analyses

#### Perioperative outcomes

**FIGURE 3:**
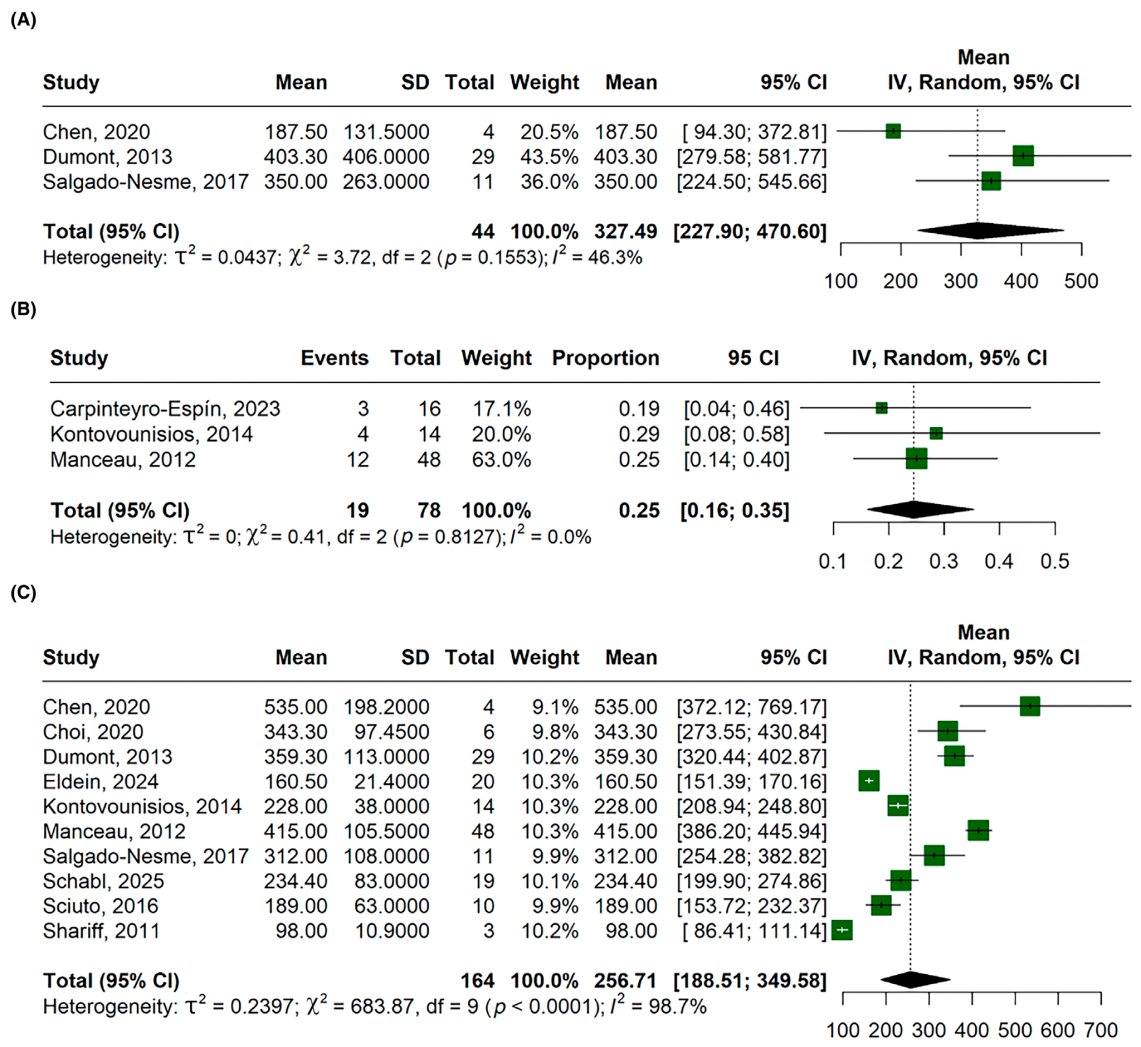
Forest plots for: (A) intraoperative blood loss (mL); (B) blood transfusion rate; (C) operating time (minutes). CI, confidence interval; IV, inverse variance.

The mean intraoperative blood loss was 327.49 mL (95% CI, 227.90–470.60; *I* ^2^ = 46.3%; Figure 3A), the blood transfusion rate was 25% (95% CI, 16%–35%; *I* ^2^ = 0%; Figure 3B) and the mean operating time was 256.71 min (95% CI, 188.51–349.58; *I* ^2^ = 98.7%; Figure 3C).

#### Postoperative recovery

**FIGURE 4:**
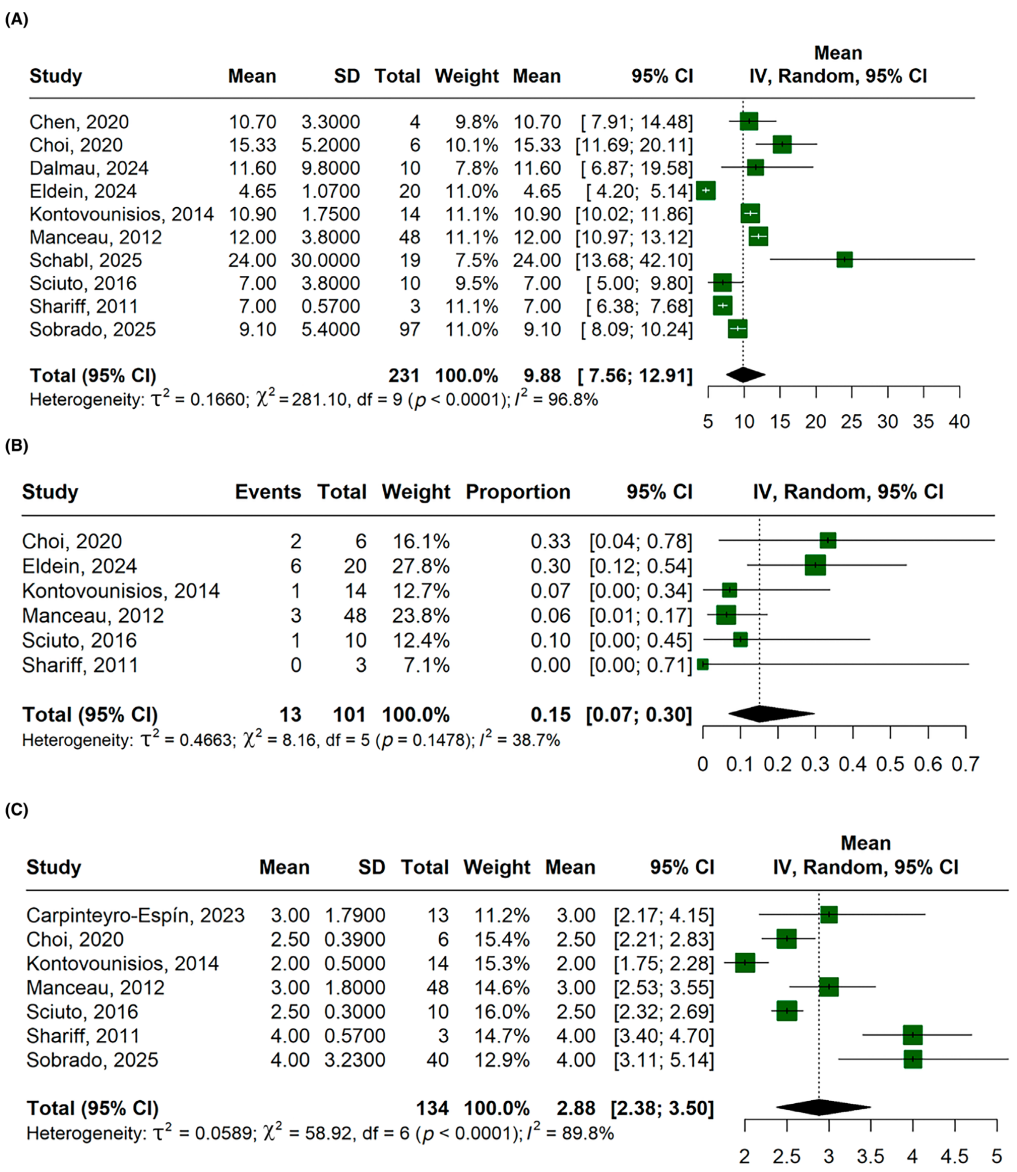
Forest plots for: (A) hospital stay (days); (B) postoperative ileus rate; (C) mean daily bowel movements. CI, confidence interval; IV, inverse variance.

The mean hospital stay was 9.88 days (95% CI, 7.56–12.91; *I* ^2^ = 96.8%; Figure 4A), the ileus rate was 15% (95% CI, 7%–30%; *I* ^2^ = 38.7%; Figure 4B) and the mean bowel movements was 2.88 (95% CI, 2.38–3.50; *I* ^2^ = 89.8%; Figure 4C).

#### Complications

**FIGURE 5:**
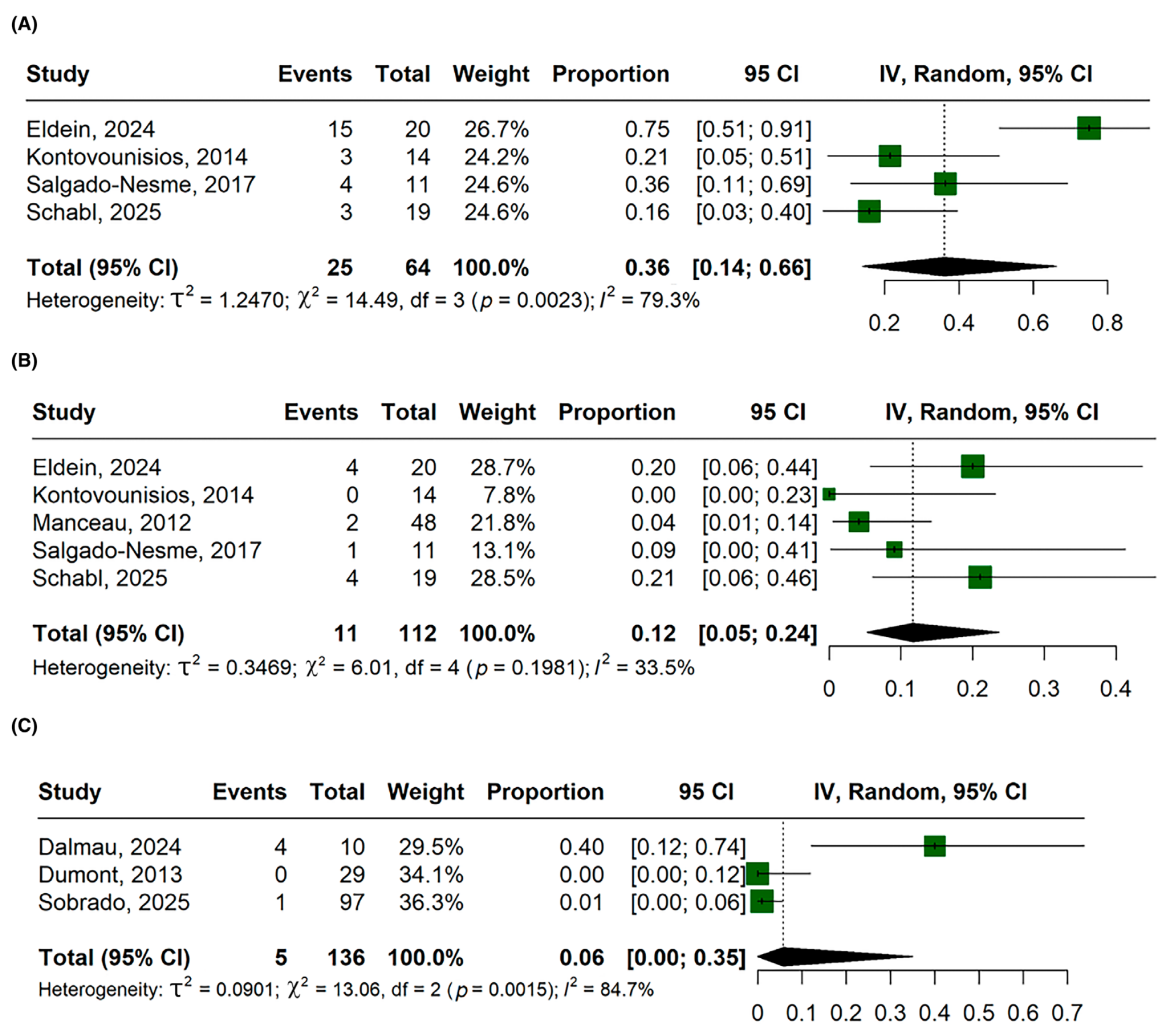
Forest plots for: (A) overall complications (Clavien–Dindo I–II); (B) major complications (Clavien–Dindo III–IV); (C) small‐bowel obstruction. CI, confidence interval; IV, inverse variance.

**FIGURE 6:**
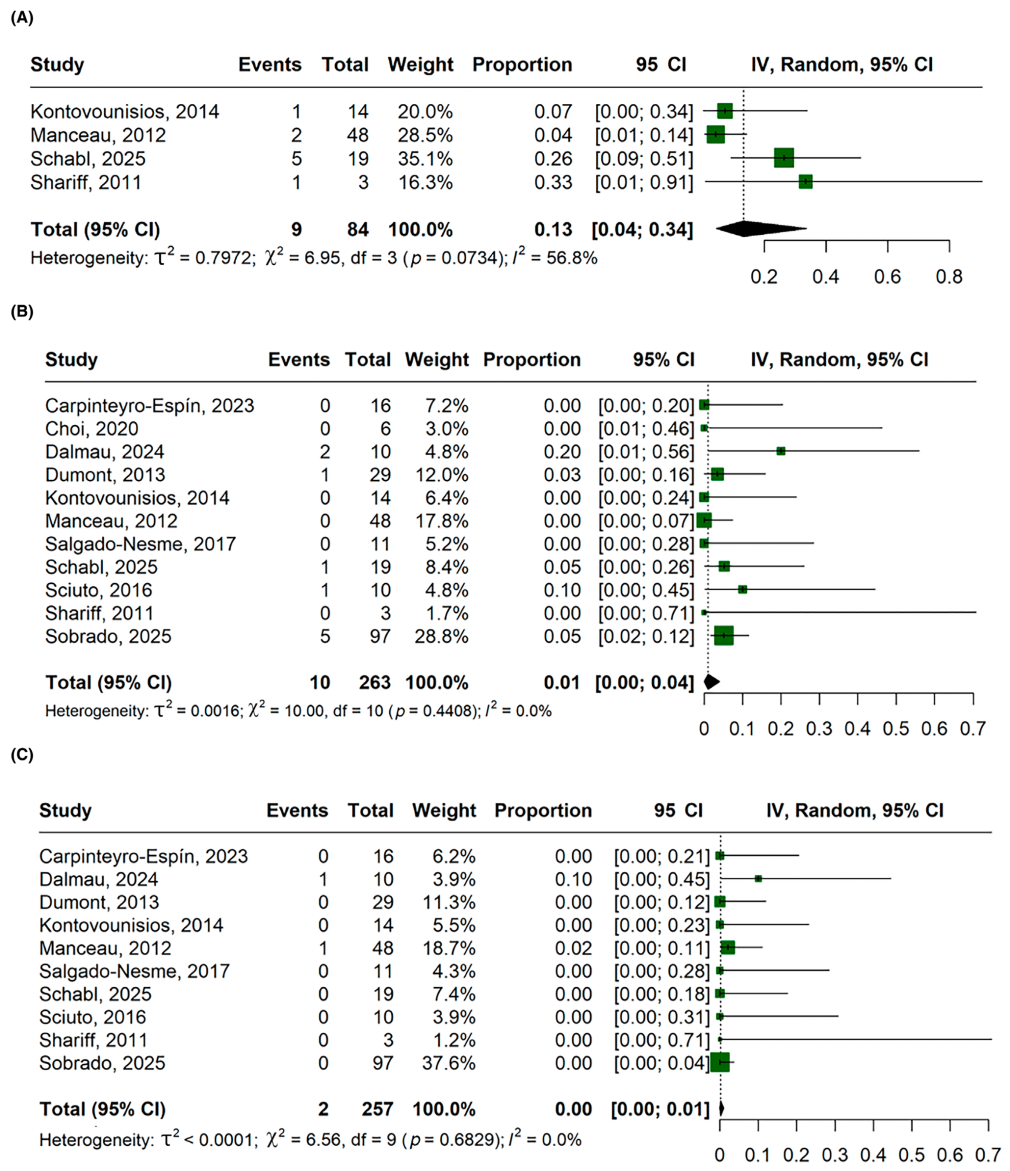
Forest plots for: (A) surgical‐site infection; (B) anastomotic leakage; (C) all‐cause postoperative mortality. CI, confidence interval; IV, inverse variance.

The postoperative complications graded CD‐I‐II rate was 36% (95% CI, 14%–66%; *I* ^2^ = 79.3%; Figure 5A), CD‐III‐IV was 12% (95% CI, 5%–24%; *I* ^2^ = 33.5%; Figure 5B), small bowel obstruction was 6% (95% CI, 0%–35%; *I* ^2^ = 84.7%; Figure 5C), SSI was 13% (95% CI, 4%–34%; *I* ^2^ = 56.8%; Figure 6A) and anastomotic leakage was 1% (95% CI, 0%–7%; *I* ^2^ = 0%; Figure 6B).

#### Mortality

The overall mortality rate was 0% (95% CI, 0%–1%; *I* ^2^ = 0%; Figure 6C). Two deaths were reported in the included studies: one hospital death in a 75‐year‐old man with significant comorbidities (ischaemic heart disease, chronic obstructive pulmonary disease and anticoagulant use) who underwent emergency surgery for diverticular bleeding, and one 82‐year‐old patient with synchronous colon tumours and bilobar liver metastases who underwent a Deloyers procedure and died postoperatively from multiorgan failure.

### Sensitivity analysis

For intraoperative blood loss, the Baujat plot identified the study by Chen et al. as the main contributor to heterogeneity, and its exclusion in the leave‐one‐out analysis reduced heterogeneity to 0%. For operating time, the Baujat plot identified Manceau et al. as the main contributor to heterogeneity; however, the leave‐one‐out analysis showed that the exclusion of any individual study affected heterogeneity. For hospital stay, the Baujat plot identified the study by Eldein et al. as the main contributor to heterogeneity; however, the leave‐one‐out analysis showed that the exclusion of any individual study affected heterogeneity. For ileus, the Baujat plot identified the studies by Eldein et al. and Manceau et al. as the main contributors to heterogeneity, and their exclusion in the leave‐one‐out analysis reduced heterogeneity to 0%.

For bowel movements, the Baujat plot identified the study by Shariffet al. as the main contributor to heterogeneity; however, the leave‐one‐out analysis showed that the exclusion of any individual study affected heterogeneity. For postoperative complications graded CD‐I‐II, the Baujat plot identified the study by Eldein et al. as the main contributor to heterogeneity, and its exclusion in the leave‐one‐out analysis reduced heterogeneity to 0%. For postoperative complications graded CD‐III‐IV, the Baujat plot identified the study by Manceau et al. as the main contributor to heterogeneity, and its exclusion in the leave‐one‐out analysis reduced heterogeneity to 0%. For small bowel obstruction, the Baujat plot identified the study by Dalmal et al. as the main contributor to heterogeneity, and its exclusion in the leave‐one‐out analysis reduced heterogeneity to 0%. For SSI, the Baujat plot identified the study by Manceau et al. as the main contributor to heterogeneity, and its exclusion in the leave‐one‐out analysis reduced heterogeneity to 0%. Sensitivity analyses are detailed in Figures S1–S18.

### Risk of bias assessment

**FIGURE 7:**
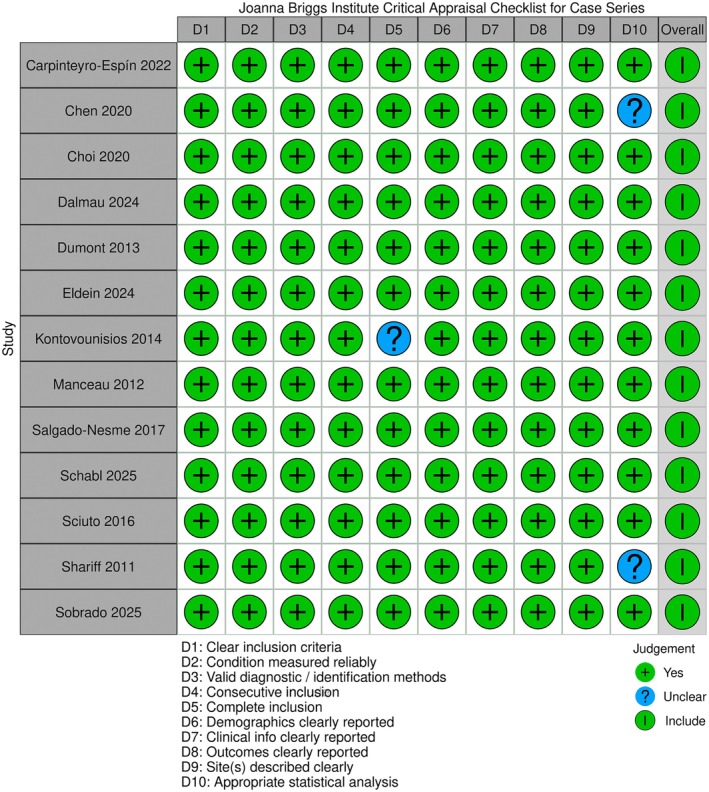
Risk of bias assessment: D, domain.

Across the 13 included case series published between 2011 and 2025, methodological quality was consistently high according to the JBI Critical Appraisal Checklist for Case Series. All studies clearly defined inclusion criteria, applied reliable methods for case identification, and comprehensively reported demographic, clinical, and outcome data. Minor uncertainties were limited to early retrospective reports, primarily due to small sample sizes or lack of inferential statistics [13, 21]. More recent studies demonstrated rigorous prospective or multicentre designs, extensive patient cohorts, and complete follow‐up [4, 19]. Overall, all studies met inclusion standards and were appraised as methodologically sound, reflecting a low risk of bias and progressive improvement in research quality over time. The risk of bias assessment is detailed in Figure 7.

## DISCUSSION

In this systematic review and single‐arm meta‐analysis of 13 studies including 287 patients undergoing the Deloyers procedure, the pooled mean intraoperative blood loss was 327 mL and the mean operating time was 257 min, with a perioperative transfusion rate of 25%. Postoperative recovery outcomes included a mean hospital stay of 9.9 days, an ileus rate of 15%, and an average of 2.9 bowel movements per day. Complication rates were 36% for CD I–II events, 12% for CD III–IV, 6% for small bowel obstruction and 13% for surgical site infection. The overall mortality rate was 0% (95% CI, 0%–1%), with two individual deaths reported in patients with significant comorbidities and advanced oncologic disease.

The Deloyers procedure is a technically complex but highly versatile option in colorectal reconstruction, especially after extended left colectomies. Its ability to preserve the colonic reservoir and enable tension‐free colorectal or coloanal anastomoses makes it a compelling alternative to two major options typically considered when a standard colorectal anastomosis is not feasible: (1) total colectomy with ileorectal anastomosis, which compromises water and electrolyte absorption; and (2) end ileostomy, which is associated with lifestyle limitations [13]. Unlike these approaches, the Deloyers technique restores bowel continuity using the native colon, maintaining function with anatomic alignment [4].

The versatility of the technique is underscored by its wide range of indications, including malignancy, diverticular disease, Crohn's disease, and redo surgeries. Most procedures in this review were performed for colorectal cancer or complicated diverticulitis. Most anastomoses were stapled; hand‐sewn reconstruction remains feasible, including in low pelvic cases, but it is technically demanding in ultra‐low settings. The Deloyers manoeuvre does not preclude either approach. The pooled mean operating time was 257 min for the entire procedure (resection plus reconstruction), not the Deloyers step in isolation. Part of this reflects careful mobilization and contralateral rotation to avoid mesenteric twist/ischaemia, while overall case complexity (e.g., extended resections) also contributes. Despite the extensive mobilization required, including detachment of the hepatic flexure and full right colon mobilization with 180° counterclockwise rotation, blood loss was modest, and transfusion requirements remained within acceptable ranges.

The mean of 2.9 bowel movements per day suggests excellent functional preservation, superior to what is often observed after ileorectal anastomosis or end ileostomy. This outcome supports the rationale for choosing Deloyers in cases where maintaining a segment of colon is feasible. Nevertheless, the expected rate of postoperative ileus, reported at 15%, may be attributable to prolonged handling and repositioning of the bowel during the procedure. Importantly, hospital stay (9.9 days) and bowel recovery timelines align with those seen in other major colorectal surgeries, suggesting that the learning curve and technical complexity do not translate into disproportionate morbidity. These observations align with a recent propensity‐matched comparison showing similar morbidity/quality of life and a trend towards fewer daily stools with Deloyers than with extended right hemicolectomy [19].

Complication rates were within expected limits, with 36% of patients experiencing minor (CD I–II) and 12% major (CD III–IV) events. Anastomotic leak occurred in 1% of patients, a particularly low rate considering the potential tension or ischaemia risks posed by the anatomic rotation of the colon. Surgical site infection was reported in 13% of patients, which aligns with the rates for procedures involving contaminated fields [ref]. Small bowel obstruction occurred in only 6%, despite concerns regarding internal herniation or torsion. Although none of the included studies explicitly reported volvulus or twisting of the neocolon, this remains a theoretical risk inherent to the 180° rotation and should be addressed through meticulous surgical technique and secure mesenteric closure. Although not systematically assessed across included studies, prophylactic appendectomy at the time of Deloyers is advisable, as the 180° rotation repositions the appendix cephalad and may obscure the clinical presentation of future appendicitis [3].

Mortality was virtually absent across the series analysed. The two reported deaths occurred in elderly patients with significant comorbidities and advanced oncological disease, reinforcing that perioperative mortality is rarely attributable to the Deloyers procedure itself. Altogether, the data suggest that when appropriately indicated and meticulously performed, this operation offers a safe and effective reconstructive option that preserves function and avoids the long‐term limitations associated with more radical alternatives.

This study represents the first meta‐analysis to evaluate the Deloyers procedure, incorporating the largest sample reported in the literature to date. However, several limitations must be acknowledged. First, this was a single‐arm meta‐analysis without a comparator group, limiting the ability to draw conclusions regarding relative efficacy or safety. Second, all included studies were observational, retrospective, and single‐centre series, making them susceptible to selection and reporting biases. Additionally, the analysis was based on aggregate data, which precluded detailed patient‐level or subgroup evaluations. Finally, the risk of publication bias remains considerable in this field, and conventional methods to assess it are not reliable in single‐arm meta‐analyses. Collectively, these limitations significantly constrain the strength and generalizability of our conclusions and underscore the urgent need for high‐quality, prospective multicentre studies with comprehensive and standardized data reporting.

## CONCLUSION

In conclusion, the Deloyers procedure is a safe and versatile reconstructive option after extended left‐sided colonic resections, particularly when colonic preservation is desired. It is associated with low anastomotic leak and mortality rates, acceptable overall morbidity, and reasonable recovery of bowel function. Despite its technical complexity, the procedure enables restoration of digestive continuity with functional outcomes likely superior to ileorectal or permanent ileostomy alternatives. Our findings support the use of the Deloyers procedure in carefully selected patients, while highlighting the need for further prospective studies to evaluate long‐term functional and oncological outcomes.

## AUTHOR CONTRIBUTIONS

**Lucas Monteiro Delgado:** Conceptualization; investigation; writing – original draft; writing – review and editing; visualization; validation; methodology; software; formal analysis; project administration; resources; supervision; data curation. **Gabriel Leal Barone:** Conceptualization; investigation; writing – original draft; writing – review and editing; visualization; validation; methodology; software; formal analysis; project administration; resources; supervision; data curation. **Giulia Luiza Garcia:** Investigation; writing – original draft; writing – review and editing; methodology; software; formal analysis; data curation. **Henrique Vaz da Mota:** Investigation; writing – original draft; writing – review and editing; resources. **Giovanna Barbaroto Pilon:** Conceptualization; writing – original draft; writing – review and editing; methodology; data curation. **Lucas Soares de Souza Pinto Guedes:** Writing – original draft; data curation; writing – review and editing. **Sérgio Mazzola Poli de Figueiredo:** Writing – original draft; writing – review and editing; conceptualization; methodology; validation; visualization; software; formal analysis; project administration; resources; supervision; data curation; investigation. **Bernardo Fontel Pompeu:** Conceptualization; investigation; writing – original draft; methodology; validation; visualization; writing – review and editing; project administration; formal analysis; software; data curation; supervision; resources. **Fernanda Bellotti Formiga:** Resources; data curation; software; formal analysis; supervision; project administration; writing – review and editing; visualization; validation; methodology; conceptualization; writing – original draft; investigation.

## FUNDING INFORMATION

This research received no specific grant from any funding agency in the public, commercial or not‐for‐profit sectors.

## CONFLICT OF INTEREST STATEMENT

F.B.F. reports being a speaker for Janssen Brazil. S.M.P.F reports honoraria from Intuitive and Distal Motion. The remaining authors declare that they have no competing interests.

## ETHICS STATEMENT

Ethical approval is not required as this study is a systematic review and meta‐analysis of previously published data.

## PATIENT CONSENT STATEMENT

Patient consent is not required as this study is based on published data.

## PERMISSION TO REPRODUCE MATERIAL FROM OTHER SOURCES

No previously published material has been reproduced in this manuscript.

## REVIEW REGISTRATION

This review was registered with PROSPERO (CRD420251135524).

## Supporting information


Data S1:

## Data Availability

Data sharing not applicable to this article as no data sets were generated or analysed during the current study.
